# CAR-T Cell Therapy and the Gut Microbiota

**DOI:** 10.3390/cancers15030794

**Published:** 2023-01-28

**Authors:** Sahana Asokan, Nyssa Cullin, Christoph K. Stein-Thoeringer, Eran Elinav

**Affiliations:** 1 Division of Microbiome and Cancer, German Cancer Research Center (DKFZ), Im Neuenheimer Feld 280, 69120 Heidelberg, Germany; 2 Faculty of Biosciences, Heidelberg University, 69120 Heidelberg, Germany; 3 Department of Internal Medicine I, Laboratory of Translational Microbiome Science, University Clinic Tuebingen, Otfried-Mueller-Strasse 10, 72076 Tuebingen, Germany; 4 Systems Immunology Department, Weizmann Institute of Science, 234 Herzl Street, Rehovot 7610001, Israel

**Keywords:** CAR-T, cancer, microbiome, microbiota, therapeutics

## Abstract

**Simple Summary:**

CAR-T cell therapy has recently revolutionized the field of cancer therapeutics, especially for hematological malignancies, and is also evolving as an experimental therapeutic option for solid tumors. Despite the groundbreaking initial response rates, nearly half of CAR-T cell treated patients have a lower response rate and experience major adverse effects. Recently, the microbiota has been suggested to constitute a contributing factor possibly impacting host antitumor CAR-T cell-mediated immune responses. As such, microbiota signatures may be harnessed to personally predict therapy response or adverse effects in optimizing CAR-T therapy to the individual. Collectively, personalized diagnostic and therapeutic utilization of the microbiota holds vast potential in achieving a safer and more efficacious CAR-T cell-based treatment.

**Abstract:**

Chimeric antigen receptor (CAR) - T cell cancer therapy has yielded promising results in treating hematologic malignancies in clinical studies, and a growing number of CAR-T regimens are approved for clinical usage. While the therapy is considered of great potential in expanding the cancer immunotherapy arsenal, more than half of patients receiving CAR-T infusions do not respond, while others develop significant adverse effects, collectively indicating a need for optimization of CAR-T treatment to the individual. The microbiota is increasingly suggested as a major modulator of immunotherapy responsiveness. Studying causal microbiota roles possibly contributing to CAR-T therapy efficacy, adverse effects reduction, and prediction of patient responsiveness constitutes an exciting area of active research. Herein, we discuss the latest developments implicating human microbiota involvement in CAR-T therapy, while highlighting challenges and promises in harnessing the microbiota as a predictor and modifier of CAR-T treatment towards optimized efficacy and minimization of treatment-related adverse effects.

## 1. Introduction

**Figure 1:**
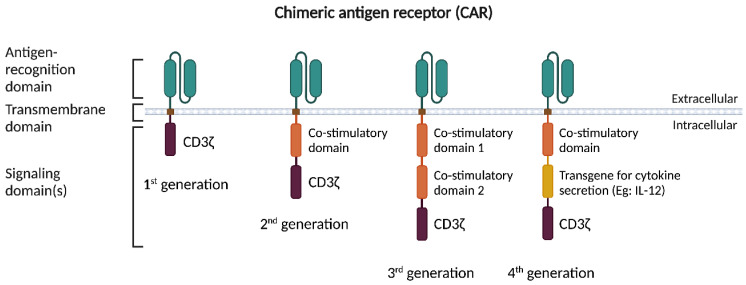
Evolution of CAR constructs. The molecular design of CARs is comprised of three regions: (i) an ectodomain comprising an antigen-binding module; (ii) a transmembrane domain as an anchor; and (iii) a signaling domain for T cell activation. First-generation CARs contain only a single signaling domain (CD3ζ). Second-generation CAR constructs include a co-stimulatory domain along with the signaling domain. Third-generation CARs are comprised of two co-stimulatory domains connected to the intracellular signaling domain. Fourth-generation CARs, also referred to as TRUCKs, have an inducible transgene construct that expresses cytokines, for instance IL-12. CAR-T, chimeric antigen receptor T; CD3ζ, cluster of differentiation 3 zeta-chain); IL-12, interleukin 12. Figure created with BioRender (biorender.com).

Chimeric antigen receptors (CARs) are genetically engineered synthetic receptors expressed in autologous T cells (CAR-Ts). CARs feature a molecular design combining an ectodomain comprising an antigen-binding module, typically a single-chain variable fragment (scFv) derived from a monoclonal antibody, and a T cell signaling module (CD3ζ: CD3 zeta-chain) connected to single/multiple intracellular signaling domain(s) of a co-stimulatory molecule such as CD28, 4-1BB, or OX40 [1,2]. Each of these elements has a distinctive function, which can be optimized by variations of these domains. Due to high CD19 expression in B cell leukemias and lymphomas, CARs targeting CD19 constitute the most widely clinically utilized CAR to date [3]. Over the years, the design of CARs has evolved considerably to enhance specificity, improve efficacy, and reduce adverse effects (Figure 1). The first-generation of CAR-T cells contained a single CD3ζ signaling domain devoid of additional co-stimulatory molecules [4,5]. These complexes were similar to endogenous T cell receptors (TCR) and specifically targeted the antigen but had modest clinical activity and a short in vivo lifespan [6,7,8]. Coupling additional co-stimulatory signaling domains (for instance, CD28, 4-1BB, or OX40) to the antigen-specific scFv led to enhanced activation, improved survival, effective expansion of the modified T cells and sustained response due to longer in vivo half-lives [9]. These second-generation CARs enabled the construction of persistent ‘living drugs’ which form the basis of current CAR-T cell therapies in clinical use. Third-generation CARs were constructed with multiple co-stimulatory signaling domains (for instance, CD3ζ-CD28-OX40 or CD3ζ-CD28-41BB) within the endodomain. A new, fourth-generation of CAR-T cell constructs combines additional T cell activity modulators with tumor-targeted effectors, such as T cells redirected for universal cytokine-mediated killing (TRUCK) CAR-T cells. These CAR-T cells express a constitutive or inducible transgenic protein expression cassette such as a transgene for cytokine secretion (e.g., IL -2) or co-stimulatory ligands to improve the antitumor activity [10,11]. 

## 2. CAR-T Cell Therapy

Through the expression of these chimeric receptors, CAR-T cell therapy has recently revolutionized the field of cancer therapeutics by redirecting autologous T lymphocytes, isolated through leukapheresis, towards a tumor-specific antigen using viral and non-viral transfection methods. CAR-T cells constitute a successful ‘adoptive cell immunotherapy’ especially suited for treatment of patients with relapsed or refractory hematological malignancies, which resulted in the U.S. Food and Drug Administration (FDA) and European Medicines Agency (EMA) approving several CAR-T cell medicines as a standard care of hematological cancers. Tisagenlecleucel (Kymriah^®^ by Novartis) was approved in August 2017 by the FDA as a therapeutic modality for treatment of patients younger than 25 years of age with relapsed or refractory B cell acute lymphoblastic leukemia (ALL) and adult patients with relapsed or refractory follicular lymphoma after two or more lines of systemic therapies [12]. Axicabtagene ciloleucel (Yescarta^®^ by Kite) was later authorized as a therapy for adult patients with relapsed or refractory diffuse large B cell lymphoma (DLBCL) after first-line treatment of chemoimmunotherapy in Europe as well as the U.S. in 2018 [13]. Since then, several CAR-T cell therapies (CD19-targeted brexucabtagene autoleucel - Tecartus^®^, lisocabtagene maraleucel - Breyanzi^®^, and B cell maturation antigen-targeted idecabtagene vicleucel - Abecma^®^, ciltacabtagene autoleucel - Carvykti^®^) have been approved by the FDA for the treatment of hematological malignancies, including lymphomas and some forms of leukemia, and most recently for the treatment of multiple myeloma [14,15,16].

**Figure 2:**
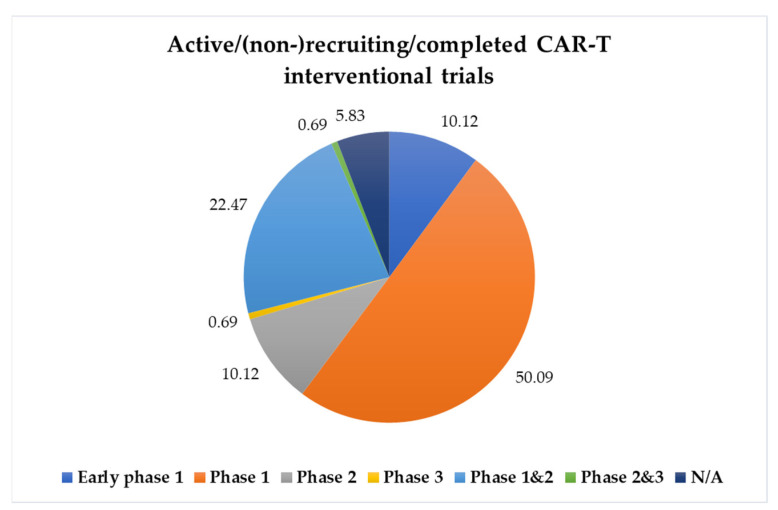
Study phase of CAR-T interventional trials. The percentage of CAR-T interventional trials that have been completed or are currently recruiting in each of the study phases. Data obtained from https://clinicaltrials.gov/; search for CAR cells; accessed on 4 January 2023. CAR-T, chimeric antigen receptor T cell.

Currently, CAR-T cell therapy is available through clinical trials for several forms of blood cancer. However, its application for solid tumors, which collectively account for ~ 90% of cancer-associated mortality, has remained challenging. Ongoing studies are exploring CAR-T cell therapy in solid tumors while primarily evaluating safety and reporting preliminary research outcomes. Over the years, such solid tumor-focused clinical trials have targeted surface proteins including carcinoembryonic antigen (CEA), Erb-B2 receptor tyrosine kinase 2 (ERBB2), epidermal growth factor receptor (EGFR), fibroblast activation protein (FAP), diganglioside (G2), human epidermal growth factor receptor 2 (Her2), interleukin 13 receptor α (IL-13Rα), L1 cell adhesion molecule (L1CAM), mesothelin, mucin 1 (MUC1), and prostate-specific membrane antigen (PSMA) [17,18,19]. However, the clinical results of CAR-T cell therapy in these solid tumor settings have been much less encouraging until some advances reported recently. For example, Jin et al. demonstrated that naturally expressed or radiation-induced expression of IL-8 enhanced intratumoral T cell trafficking [20]. Indeed, IL-8 upregulation at the tumor invasion front has been demonstrated in several types of human cancers [21]. Consequently, tumor-produced IL-8-guided CAR-T cells facilitated migration into tumors, thereby prompting an enhanced antitumor response in solid tumors [20]. Additionally, multiple novel target antigens are being investigated in preclinical and clinical trials across different types of cancers [22]. Currently, 995 CAR trials are ongoing (results from https://clinicaltrials.gov/; search for CAR cells; accessed on 4 January 2023): nearly 49% of the trials are currently recruiting, while 5% of the trials have been completed. Among the completed/recruiting/active, not-recruiting interventional trials, approximately 10% of the studies are in early phase 1; 50% are in phase 1; 10% are in phase 2; 0.7% are in phase 3; 22% are in both phase 1 and 2; 0.7% are in phase 2 and 3; while in nearly 6% of studies the information on current trial phase was not identifiable. The graphical representation of the completed/recruiting/active, not-recruiting CAR-T trials in different study phases has been illustrated in Figure 2 and has been summarized in Table S1. 

## 3. Toxicity Associated with CAR-T Therapy

In addition to the 50% of CAR-T-treated patients who do not respond to therapy or relapse after therapeutic intervention, a significant number of patients experience severe adverse effects including cytokine release syndrome (CRS) and immune effector cell-associated neurotoxicity syndrome (ICANS). If left unchecked, these lead to adverse outcomes that do not allow for therapy responses, in part due to significant morbidity and mortality. 

CRS is marked by overall symptoms of fever, exhaustion, anorexia, myalgia, and arthralgia, which can further progress to more severe forms of the syndrome including cardiac conditions (arrhythmia, tachycardia) and respiratory (tachypnea) and multi-organ failure [23,24,25]. CRS is driven by the release of inflammatory cytokines, including IL-2, IL-6, IL-10, IFNγ, and TNFα [26,27], as a direct consequence of the response to CAR-T cells and additional surrounding immune cells, leading to an overall hyperinflammatory response [26,27,28]. Immune effector cell-associated neurotoxicity syndrome (ICANS) is characterized by initial symptoms relating to impaired cognition and overall confusion including aphagia, lethargy, and delirium [24,29,30]. Over time, ICANS can progress to seizures, coma, and cerebral edema [31,32]. Blood–brain barrier disruptions, influx of cytokines into the central nervous system (CNS), and microglial as well as myeloid activation within the CNS are considered contributors to ICANS in relation to CAR-T infusion and cell migration [32,33,34]; however, the exact causes of ICANS are not fully understood. CRS can be treated by symptomatic treatment, IV hydration and in higher-graded CRS with corticosteroids and anti-cytokine treatments such as tocilizumab targeting IL-6. In more serious cases of ICANS, anti-epileptics may be necessary to manage seizures [35]. 

CAR-T therapeutic efficacy is also altered by potential off-target effects of the CAR-T cells. In CAR-T therapies targeting CD19-expressing malignant cells, such off-target effects include infection susceptibility driven by non-malignant B cell aplasia and resultant hypogammaglobulinemia. In patients receiving CD19-targeted CAR-T cells to treat DLBCL, more than half of those enrolled in a one-year study developed infections, with the majority of those attributed to bacteria [36]. Strikingly, many of the bacteria identified in blood-stream infections in these patients such as *Escherichia*, *Pseudomonas*, and *Staphylococcus* notably originate in the gut prior to systemic translocation [36,37,38].

## 4. Microbiota Involvement in CAR-T Response and Toxicity

Over the last decade, a multitude of preclinical and clinical studies have demonstrated an interplay between the intestinal commensal microbiota and the mammalian immune system development and function [39]. Moreover, the intestinal microbiota was suggested to correlate and even modulate responses to anticancer therapeutics including chemotherapy, radiotherapy, immune checkpoint blockade, and adoptive cellular therapy, potentially by impacting host antitumor immune responses [40,41,42,43]. For example, Paulos et al. demonstrated that microbiota translocation augmented TLR4-mediated activation of the immune system, thereby enhancing the efficacy of adoptively transferred self/tumor-specific CD8^+^ T cells [44]. Likewise, allogeneic hematopoietic cell transplantation (allo HCT) was correlated to gut microbiota changes induced by dietary and antibiotic exposure [45,46], including an expansion of *Enterococcus* associated with a higher risk of graft-versus-host disease (GVHD)–related mortality [28,45,47]. Conversely, *Eubacterium limosum* has been associated with a decrease in cancer relapse/progression after allo HCT in patients with hematologic malignancies [48]. Emerging data also suggest that the gut microbiota may impact immune checkpoint blockade therapies, targeting programmed cell death protein 1 (PD-1) and cytotoxic T lymphocyte-associated protein 4 (CTLA-4), as is extensively reviewed elsewhere [28,49]. Briefly, a variety of taxa including *Bacteroides*, *Akkermansia, Faecalibacterium*, and *Clostridiales* spp. have been identified in murine studies to be associated with PD-1 therapy and its ligand PD-L1 and suggested to enhance the overall antitumor efficacy of checkpoint blockade [49,50,51]. *Bacteroides fragilis* and *Bacteroides thetaiotaomicron* were shown to enhance the CTLA-4 inhibitor efficacy in mice [50,51], collectively indicating a potential role for the microbiota in altering responses to CAR-T cell therapy [52]. 

Similar microbiota impacts were recently suggested to impact CAR-T cancer immunotherapy efficacy. In a first human study, Smith et al. analyzed the fecal microbiota composition of patients receiving second-generation CD19-targeted CAR-T therapy for treatment of B cell malignancies, hypothesizing that the microbiota would have associations with treatment efficacy and toxicity. Baseline stool samples prior to CAR-T therapy were heterogenous for bacteria at the phylum level and present with a decreased Shannon index for alpha diversity as compared to healthy controls. At the genus level, the patient microbiota significantly differed from that of healthy controls [53]. Given that antibiotics are commonly administered to treat secondary infections in patients undergoing anticancer therapies, antibiotics exposure and associated dysbiosis were suggested to adversely affect the overall clinical outcome of immunotherapies [54]. Indeed, Smith et al. also noted that 60% of their patient cohort received antibiotics and 20.6% of the cohort specifically received broad-spectrum antibiotics such as piperacillin/tazobactam, imipenem/cilastatin, and meropenem (PIM) that target anaerobic gut commensal bacteria. PIM exposure prior to CAR-T therapy correlated with worse overall survival and progression-free survival; notably, PIM correlated with a more aggressive disease and a higher lactate dehydrogenase, which is a biomarker of a higher tumor burden. Patients receiving any antibiotics in the weeks preceding CAR-T therapy initiation displayed increased incidence of ICANS. Specifically, exposure to PIM also correlated with a higher ICANS in non-Hodgkin lymphoma (NHL), but not in ALL. CRS was not shown to be correlated with PIM exposure. In all, multiple bacterial species were associated with the absence of toxicity, but microbes associated with toxicity were unidentifiable by a linear discriminant analysis effect size (LEfSe). Complete response rates at day 100 after CAR-T cell infusion were also correlated with a higher abundance of certain taxa, specifically the class Clostridia, further indicating that the gut microbiota may be playing a role in modulating the efficacy of CAR-T therapy. Overall, Smith et al. concluded that antibiotic exposure and its alteration of the gut microbiota prior to CAR-T therapy likely plays a role in its antitumor efficacy and toxicity [53].

Another recent study by Hu et al. [55] investigated CAR-T toxicity in relapsed/refractory multiple myeloma (MM), NHL, and ALL patients receiving second-generation therapy related to changes in the gut microbiota. Microbiota changes were longitudinally monitored throughout CAR-T delivery by stool sampling prior to CAR-T infusion, during CAR-T infusion but prior to development of CRS, during active CRS, and up to fourteen days after CAR-T infusion. Severe CRS was associated with a decreased abundance of *Bifidobacteria*. Alpha diversity as indicated by the Shannon index significantly decreased after CAR-T infusion and was further associated with an increase specifically in the abundance of *Actinomyces* and *Enterococcus* genera. Furthermore, *Prevotella*, *Collinsella*, *Bifidobacterium*, and *Sutterella* spp. were more abundant in patients experiencing complete response versus partial response [55]. Likewise, Smith et al. reported that patients with a higher abundance of certain bacterial species such as *Ruminococcus, Bacteroides,* and *Faecalibacterium* had a better response to CAR-T cell therapy [53]. Overabundance of *Enterococcus faecium*, post-antibiotics treatment in CAR-T patients, was further negatively correlated with treatment response [56]. In all, antibiotic exposure and the subsequent alteration of the gut microbiota associates with increased toxicities including CRS and ICANS, and with worsened CAR-T responses.

Importantly, these antibiotic effects may represent a causal impact of the antibiotics-perturbed microbiota on CAR-T therapy-related endpoints, or alternatively a reverse causality, in which antibiotics-treated patients present in an a priori clinically worse state predisposed to altered CAR-T therapy responsiveness. To untangle these possibilities, a recent study (Stein-Thoeringer et al., in press) [57] followed a large cohort of lymphoma patients receiving second-generation CD19-targeted CAR-T cells in Germany and the U.S. Like previous studies, an association was noted between an exposure to antibiotics prior to CAR-T cell infusion and an increased prevalence of cancer relapse or disease progression and a decrease in overall survival. However, wide spectrum antibiotics-treated patients suffer from an a priori worse disease state and increased tumor burden, likely accounting for their decreased CAR-T therapy responsiveness. Excluding these patients allowed for the detection of microbiota signatures strongly correlating with CAR-T responsiveness. Moreover, a cross-country evaluation of non-wide spectrum antibiotics-treated patients enabled a machine learning microbiome-based prediction of treatment outcomes, and the identification of *Bacteroides*, *Ruminococcus*, *Eubacteria*, and *Akkermansia* spp. as major potential drivers of therapy responsiveness. 

**Figure 3:**
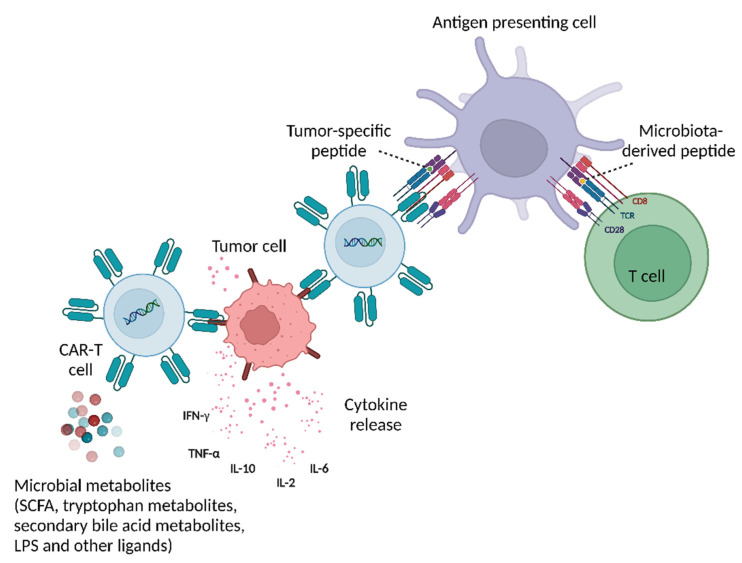
Microbiota involvement in CAR-T therapy efficacy and toxicity. Gut microbiota-derived peptides and metabolites exert their influence on both T cells as well as CAR-T cells, which can be further modulated by modification of diet and/or administration of antibiotics. The abundance of certain species in the gut microbiome facilitates therapeutic efficacy while dysbiosis leads to adverse effects including CRS and ICANS, increased tumor relapse or disease progression, and decreased overall survival. CRS, cytokine release syndrome; ICANS, immune effector cell-associated neurotoxicity syndrome; SCFA, short chain fatty acids; LPS, lipopolysaccharide, IFN-γ, interferon gamma; TNF-α, tumor necrosis factor alpha; IL-10, interleukin 10; IL-2, interleukin 2; IL-6, interleukin 6, CAR-T, chimeric antigen receptor T; TCR, T cell receptor; CD 28, cluster of differentiation 28; CD 8, cluster of differentiation 8. Figure created with BioRender (biorender.com).

Microbiota modulation of CAR-T treatment can be also driven by microbially secreted metabolites (Figure 3). A recent in vivo study demonstrated that CAR-T therapy modified to possess a receptor tyrosine kinase-like orphan 1 (ROR1) receptor induced a significant decrease in tumor volume and weight in a subcutaneous mouse model of pancreatic cancer featuring ROR-1-expressing Panc02 cells. The effect of these ROR1 CAR-T cells was further modulated with an addition of the short chain fatty acids (SCFA) butyrate and pentanoate [58]. Indeed, microbial-derived SCFA may favorably impact multiple immunotherapies, through a variety of mechanisms including enhanced TNFα and IFNγ effector responses, as well as upregulating anti-inflammatory T regs and CD8+ T cell functions while minimizing pro-inflammatory macrophage, dendritic cell, and Th1/Th17 activities [58,59]. Whether similar impacts would be observed in the human setting, and whether other bioactive metabolites participate in such interactions merits future studies.

## 5. Therapeutic Microbiota-Mediated Modulation of CAR-T Efficacy

Growing evidence delineates that modulation of the intestinal microbiota may impact the outcome of a variety of microbiota-contributed diseases, including cancer and immunotherapy. For example, two pilot first-in-human clinical trials recently provided evidence that melanoma immunotherapy responder-derived fecal microbiota transplantation (FMT) in combination with anti-PD-1 may benefit a subset of patients with PD-1-refractory melanoma [60,61]. 

As noted above, microbiota-modulated bioactive metabolites (termed ‘postbiotics’), such as SCFA, may enhance the antitumor action of cytotoxic lymphocytes and CAR-T cells [58]. These effects could be carried out by metabolic and epigenetic remodeling of CAR-T cells, driving increased expression of effector molecules such as CD25, IFNγ, and TNFα in syngeneic murine melanoma and pancreatic cancer models [58], or by SCFA binding to the G-protein-coupled receptor GPR109A on T cells, promoting T cell killing after high antigen stimulation [62]. Other microbial-derived metabolites, signaling through aryl hydrocarbon receptors (AhR), may also play a role in contributing to CD8+ T cell exhaustion by upregulating inhibitory receptors and downregulating cytokine production, thereby altering the ability of T cells to kill tumor cells [63,64]. Supplementation or inhibition of such microbially secreted bioactive metabolites may potentially be used to reinvigorate the immune response.

Modulating dietary content and timing can alter microbiota community structure and related metabolite secretion profiles, thereby impacting host physiology including CAR-T therapy responsiveness and adverse effect profiles. Such ‘personalized nutrition’ approaches have been shown to impact glycemic response outputs in a reproducible, microbiota-dependent manner [65,66]. For instance, a diet high in fibers induces the production of butyrate, propionate, and acetate, which have been linked to anti-inflammatory pathways in mouse cancer models [67,68,69]. As previously shown in non-cancer contexts, monitoring the glucose levels of hundreds of individuals showed highly variable responses to similar meals and predicting this outcome and establishing an optimal diet using machine learning effectively altered the post-prandial glycemic responses in individuals; thus, it may be feasible to integrate personal microbiota and host features by artificial intelligence and machine learning tools in harnessing dietary responses of the individual towards optimization of CAR-T therapy responses [49,65,70]. Targeted suppression of microbes associated with CAR-T therapy non-responsiveness and higher incidence of CRS may constitute another attractive modality in optimizing treatment. Rationally designed bacteriophage combinations, for instance, have been recently utilized to specifically suppress intestinal pathogens associated with inflammatory bowel disease and therefore may serve as a potential targeted method in suppressing pathobionts in other microbiome-contributing clinical conditions [71]. These modalities merit further consideration in CAR-T-treatment contexts.

## 6. Limitations and Challenges 

Defining the causal effects of the microbiota and associated secreted bioactive compounds, rather than merely relying on associations and correlations, remains a major challenge in microbiota-associated research. Establishing mechanisms proving such causal effects in the CAR-T therapy context will likely require further in vitro and in vivo research, including studies utilizing animal models of cancer and CAR-T therapy. Transferring defined microbiota configurations from human CAR-T responders and non-responders into cancer-bearing germ-free mice would enable such elucidation of the causal contribution of microbial consortia and their bioactive metabolites to treatment responsiveness [49]. Inter-individual microbiota variability represents another formidable challenge in identifying reproducible and generalizable microbes and bioactive compounds impacting CAR-T therapy and adverse effects across large patient populations. Indeed, both Smith et al. and Hu et al. report high variability in microbiota populations between CAR-T-treated patients, characterized by the dominance of different phyla [53,55]. Similarly, varying taxa have been associated with impaired immunotherapy responsiveness in different trials, indicating that defining a generalizable CAR-T therapy optimizing microbial signature will be difficult to achieve [72,73,74]. This further highlights the need to include multicentric clinical trials with high-quality training and validation sets in identifying CAR-T therapy-related microbiota signatures [45,75]. Such efforts will be aided by the use of artificial intelligence technologies, in utilizing heterogenous patient-derived data towards actionable conclusions [65]. Of note, whole microbiota transfers into CAR-T-resistant individuals may optimize responsiveness and even convert non-responders to responders. Such treatment, however, poses risks of introducing potentially harmful bacteria into patients who are already severely immunocompromised, while inducing off-target effects, barrier disruption, and even sepsis [49,76]. In the long term, identification of defined consortia mediating such favorable effects may offer a safer, more reproducible, and universal treatment option. Finally, the focus of this review has been on the influence of the bacterial microbiota in patients receiving CAR-T therapy. The microbiota additionally includes viruses, fungi, and parasites that could all potentially influence the efficacy of CAR-T therapy. Studying the impacts of these insufficiently explored commensal kingdoms holds vast potential for the development of an even larger set of therapeutic microbiota-related modulations.

## 7. Conclusions

CAR-T therapy has revolutionized the treatment of hematological malignancies and overall advanced our understanding of modern cancer therapeutics. Determining novel methods to enhance CAR-T efficacy and responsiveness is of utmost concern. The human microbiota has shown profound influence as a modulator of immunotherapy response. Here, we review the recent reports that microbiota features such as abundance of various species correlate with CAR-T toxicity and overall response. Furthermore, the potentially confounding nature of antibiotic usage upon microbiota as related to CAR-T therapy should be further studied and defined.

## Supplementary Materials

The following supporting information can be downloaded at https://www.mdpi.com/article/10.3390/cancers15030794/s1, Table S1: Summary of the latest CAR-T interventional trials in different study phases.

## Abbreviations

AhR	aryl hydrocarbon receptors
ALL	acute lymphoblastic leukemia
allo HCT	allogeneic hematopoietic cell transplantation
CAR	Chimeric antigen receptor
CD3ζ	cluster of differentiation 3 zeta-chain
CEA	carcinoembryonic antigen
CNS	central nervous system
CRS	cytokine release syndrome
CTLA-4	cytotoxic T lymphocyte-associated protein 4
DLBCL	diffuse large B cell lymphoma
EGFR	epidermal growth factor receptor
EMA	European Medicines Agency
ERBB2	Erb-B2 receptor tyrosine kinase 2
FAP	fibroblast activation protein
FDA	U.S. Food and Drug Administration
FMT	fecal microbiota transplantation
G2	diganglioside
GVDH	graft-versus-host disease
Her2	human epidermal growth factor receptor 2
ICANS	immune effector cell-associated neurotoxicity syndrome
IFN-γ	interferon gamma
IL	interleukin
IL-13Rα	interleukin 13 receptor α
L1CAM	L1 cell adhesion molecule
LEfSe	linear discriminant analysis effect size
LPS	lipopolysaccharide
MM	multiple myeloma
MUC1	mucin 1
NHL	non-Hodgkin lymphoma
PD-1	programmed cell death protein 1
PSMA	prostate-specific membrane antigen
ROR1	receptor tyrosine kinase-like orphan 1
SCFA	short chain fatty acids
scFv	single-chain variable fragment
TCR	T cell receptors
TNF-α	tumor necrosis factor alpha
TRUCK	T cells redirected for universal cytokine-mediated killing
